# Cases of Fracture of the Neck of the Humerus

**Published:** 1827-07

**Authors:** 

**Affiliations:** St. Thomas's Hospital.


### FRACTURE OF THE HUMERUS.

*Cases of Fracture of the Neck of the Humerus, treated by* Mr. Travers, *at* St. Thomas's Hospital. *With Remarks, by* J. Hainworth, Surgeon.

The term "neck of the os humeri" is generally understood, in surgical language, to apply to that portion of the bone which is below the head and above the insertion of the pectoralis major, latissimus dorsi, and teres major muscles. A fracture in this situation has long been set down as the most difficult to detect of all the fractures of the humerus, and has not unfrequently been mistaken for a fracture of the neck of the scapula, or a dislocation of the head of the humerus into the axilla. From the description, however, of this accident given in one of the latest and most esteemed works on this subject, we should be led to imagine that the diagnosis was easy, the point which constitutes the difficulty being only incidentally mentioned; and, but for a fortunate accident occurring at the moment of writing the description, it would in all probability have been passed over without the slightest notice. The author states, that "in this accident the head of the bone remains in its place, but the body of the humerus sinks into the axilla, where its extremity can be felt; and it draws down the deltoid muscle, so as to *lessen the roundness* of the shoulder." If these appearances were presented immediately after the accident, there could be no impediment to prevent the adoption of the mode of ascertaining the injury subsequently recommended,—viz. by grasping the head of the humerus, and rotating the body of the bone, when the head will be found to remain unmoved. The author, however, proceeds to give the notes of a case of this kind, which was brought to Guy's Hospital at the time he was writing the above description; and here it was observed, among other symptoms, that "the tension which succeeded the accident filled up the hollow which was at first produced by the fall of the deltoid muscle." By comparing these two accounts, we shall be inevitably led to the conclusion that the general description was drawn either from considering the appearances which a fracture of the neck of the humerus would present in the dead subject, or that it was derived from the appearances presented several days after the infliction of the injury. In drawing this conclusion, we are aware that it may appear presumptuous to question the accuracy of so great an authority as Sir Astley Cooper; but when the case given us by himself is directly at variance with his previous statements, and when this case is corroborated in every point by others of a similar kind, it surely becomes our duty to look with a suspicious eye on an account which differs so widely from carefully noted histories, and to direct the professional public to an impartial investigation of the subject for themselves. With this view only have these remarks been drawn up, founded on an attentive observation of the subjoined cases.

The source of all the difficulty which has been experienced by surgeons in endeavouring to detect this fracture, appears to consist in the great extravasation of blood which takes place immediately after the accident, in consequence of the very severe contusion sustained by the soft parts about the shoulder, and particularly by the deltoid muscle, and in the consequent inflammation and effusion of lymph and serum; the injury being commonly inflicted by a fall on the point of the shoulder. This extravasation and effusion produce enormous swelling and distention of the upper part of the limb, so that the natural rotundity of the shoulder is much increased, and the head of the humerus cannot by any means be felt; the whole mass becomes so consolidated together that no crepitus can be perceived on moving the limb, which may be readily done in every direction by applying slight external force, although very acute pain is excited. The patient himself is utterly incapable of making the least exertion, and allows the whole shoulder to droop, so as in some instances to have given rise to the mistaken notion that the neck of the scapula had been fractured. This drooping of the shoulder cannot be considered as diagnostic in this case, as it is a symptom common to almost every injury of the shoulder and upper part of the arm, from a simple contusion to the most complicated mischief which can be inflicted. Partly from this drooping, and partly from the induration of the axilla by the extension of the extravasation, partly also from the displacement inwards of the lower fractured portion, such a protuberance has appeared in the axilla as to lead to the belief that the head of the humerus was dislocated; and, in the absence of crepitus, this opinion appears plausible enough; but, in addition to the mobility of the limb in all directions, the slightest inquiry as to the mode in which the accident happened will at once lead to the certainty that such a displacement of the head of the bone is impossible; for, as before observed, the point of the shoulder has, in almost every case, received the full force of the fall.

This account, which applies to the first six or eight days after the accident, will sufficiently explain the difficulty experienced by surgeons of all periods in detecting at once the nature of the injury; and at this stage it is, in fact, almost impossible to give a decided opinion as to the existence of fracture or otherwise: neither is it of so much importance to arrive at certainty, for, in the inflamed and swollen state of the limb, distending the integuments to the utmost, no surgeon would think of applying bandages and splints without first endeavouring to reduce the inflammation; for otherwise he would almost infallibly produce gangrene. The circumstances already pointed out, when connected together, will excite a strong suspicion that the neck of the humerus is fractured; and this is all that the surgeon can require to govern his conduct, as he has a sufficient number of facts to satisfy him that the head of the bone is not dislocated, and he knows that, by confining the patient in bed, supporting the injured arm on a pillow, and by the frequent application of leeches and the continued employment of cold lotions, the inflammation will be subdued, and the extravasated blood, and effused serum and lymph, will be gradually absorbed in the course of seven or eight days, leaving the shoulder in the state described by Sir A. Cooper as occurring immediately after the accident, when the most satisfactory assurance may be obtained of the actual state of the bone. Not only will the effused fluid be removed about this time, but the deltoid will have become so much reduced in size by the process of interstitial absorption, that the bone will be found to lie almost immediately beneath the integument, and there is not the slightest difficulty in ascertaining that the head of the humerus occupies its place in the glenoid cavity: it may also be readily grasped between the fingers, and will be found to remain unmoved while the body of the bone is rotated; crepitus will also be readily perceived, though somewhat modified by the deposit on the fractured extremities; and, in most instances, the point of the lower portion will be felt in the axilla. This, of course, refers to the fracture above the insertions of the pectoralis major, latissimus dorsi, and teres major muscles: when below these, and above the insertion of the deltoid, the point of the upper portion will be drawn inwards, and the lower will project beneath the deltoid.

Little is required to be said on the treatment of the fracture at this stage. The limb must be extended by fixing the shoulder, and using the forearm as a lever to draw down the body of the humerus; a well-padded splint may then be placed on the inner and outer side of the arm, and fixed with a carefully applied roller; a large pad must now be placed in the axilla, binding it there by a roller crossed over the shoulder on the injured side, and carried under the opposite shoulder; then, bringing the elbow to the side by means of a bandage passed round the body, the lower portion is prevented from being again displaced; the forearm may now be supported in a sling in a supine position, to relax the biceps muscle. On removing the apparatus at the end of three or six weeks, according to the age of the patient and the powers of his constitution, the fracture will generally be found firmly reunited, and a few weeks will suffice to restore the limb to its former vigour.

*Case of Fracture of the Neck of the Humerus, in which the lower portion projected a little into the Axilla, the Patient regaining full power over the Limb.*

June 13th, 1825.—Hugh Jullion, æt. thirty-six, was returning home last evening in a gig, in a state of intoxication, when the horse took fright and overturned the vehicle, throwing the man with considerable violence to the ground, the upper part of his shoulder receiving the full force of the fall. The arm was rendered useless, and, on application to a surgeon, he was told that it was broken, and advised to go to the hospital. On admission, about fifteen hours after the accident, the soft parts covering the shoulder-joint were so much swollen, and rendered so tense, that an accurate idea of the nature of the accident could not be formed. Some crepitus could be occasionally perceived, though very indistinctly; and although some irregularity was found in the axilla, yet it was sufficiently clear that it did not contain the head of the humerus, and the elbow was easily brought to the side. The patient complained much of the pain, which the examination aggravated considerably.

Leeches were applied, and occasionally repeated; spirit lotion. The patient was kept in bed several days, supporting the arm by a pillow. Aperient medicines were given; and, as he complained much of pain and want of sleep, he was ordered to take Extr. Hyosc. gr. v. omni nocte; which on the 18th was omitted, and Pil. Sapon. cum Opio gr. v. substituted.

The swelling and tension subsided very gradually. On the 23d, the stellate bandage was applied, with a pad in the axilla; a splint, reaching from the shoulder to the external condyle, applied; the elbow confined to the side, and the forearm supported by a sling.

July 23d.—The fracture has firmly united; the lower point of bone projects slightly in the axilla; the arm is stiff, but permits of motion in every direction.

August 10th.—The muscles of the arm, which had wasted considerably, have now nearly regained their former size. The movements of the joint are perfect, and performed with tolerable facility.

*Case of Fracture of the Head and Neck of the Humerus, in which firm Union took place, leaving the Movements of the Joint perfect.*

October 11th, 1826.—Joseph Miller, æt. eighteen, a strong healthy lad, was early this morning thrown from his horse, falling upon the point of the right shoulder. He was admitted nine hours after the accident, with immense swelling and great tension of the whole shoulder, and particularly of the deltoid muscle, the integuments covering them being very hot, and much discoloured; the shoulder is considerably depressed; there is no tumor in the axilla; and though the boy has lost all power over the limb, yet the elbow can be carried to the side, and the hand raised to the head. In consequence of the enormous swelling, neither crepitus nor rotation of the head of the humerus can be perceived. He complains of great pain, violently increased by moving the arm, particularly in rotation.

**Ordered to keep his bed, supporting the arm by a pillow. Apply Hirudines xx., and afterwards a poultice to encourage the bleeding.—Spirit lotion.**

The leeches were occasionally repeated, and in the course of a few days the swelling had in some degree subsided, and the pain was much alleviated.

18th.—The swelling is somewhat reduced, but the shoulder is still misshapen; there is about double the distance between the front and back part of the head of the os humeri, as compared with the opposite shoulder. On careful examination, a fissure may be felt, though indistinctly, extending from below, upwards and forwards, through the head of the bone. On rotating the arm, and fixing the head, a crepitus is perceived, as of one flat surface thrown off another, and again striking it: this motion excites great pain.

A pad was now placed in the axilla; a stellate bandage applied; a splint placed on the outer side of the arm, and the elbow confined to the side, supporting the forearm in a sling. In the course of a few days, he became much easier, and the swelling entirely disappeared.

November 20th.—The apparatus was removed. The deltoid muscle has wasted considerably, and the state of the bone can be both seen and felt. The space from the back to the front of the head is about two-thirds wider than in the sound limb; anteriorly is an irregular projection of bone, forming about the lower one-fourth of the head of the os humeri, and from this a slight ridge of callus may be traced, extending obliquely downwards and backwards a short distance into the shaft of the bone. From long confinement the arm is nearly fixed to the side.

Passive motion was employed daily, and also friction, and by the 11th of December the deltoid and other muscles had regained about half their bulk, and the lad could use the arm tolerably well in all directions.

*Case of Fracture of the Cervix Humeri, in which an opportunity was afforded of examining the Limb six Months after the Accident.* — the party having been so kind as to die.

January 1st, 1818.—Patrick Bryan, æt. fourteen, slipped in walking, and fell upon the point of the shoulder, about an inch below the acromion, against the edge of a step. He states that he was stunned by the fall, and has since lost all command over the arm. On his admission, about twenty-four hours after the accident, very considerable effusion, and consequent tension, existed on the anterior part of the joint, which was much more rounded than natural; there was no depression below the acromion; neither was there any fullness in the axilla. Grasping the head of the humerus between the thumb and finger before and behind the acromion, and rotating the arm, a distinct crepitus is perceived; and pressure upon this part occasions much pain.

Applic^r Hirudines xij. et postea Lotio Alba c. Spir. Vinos.—Sumatur Haustus Purgans.

January 6th.—Tension and pain much relieved. The stellate bandage, with a pad in the axilla, was now applied, and the arm supported in a sling.

The tension gradually subsided, and on the 31st the bandage was left off.

February 11th.—He was discharged, all the motions of the shoulder-joint being performed with nearly as much freedom as before the accident occurred.

In the month of June following, he was admitted with a very severe laceration of the hand and forearm, inflicted by a wool-carding machine, for which amputation was performed. The lad, however, died in two days; and, on examining the humerus, it was found to have been fractured a little below its head. It had firmly united, giving to the bone, however, somewhat of the shape of the os femoris.

